# Commentary: Joint effect of sleep duration and sleep quality on self-rated health among Canadian adults: estimating relative excess risk due to interaction from a nationwide survey

**DOI:** 10.3389/fpubh.2025.1725525

**Published:** 2026-01-15

**Authors:** Juma Rahman, Bapon Fakhruddin

**Affiliations:** 1 University of Auckland, Auckland, New Zealand; 2 Research & Evaluation Office (REO), Auckland, New Zealand; 3 CODATA, International Science Council, Paris, France

**Keywords:** additive interaction assessment, interaction, relative excess risk due to interaction, sleep duration, sleep quality, statistical approaches

## 1 Introduction

Sleep duration and sleep quality are well-established determinants of overall health. Extensive evidence showing that inadequate or poor-quality sleep increases the risk of cardiovascular disease, metabolic disorders, mental-health problems, and all-cause mortality (1–3). Additionally, recent umbrella reviews and population-based studies confirm that both short sleep and poor sleep quality independently and jointly contribute to adverse physical and psychological outcomes, including depression, anxiety, chronic pain, and reduced subjective wellbeing (4–6).

In light of this, we express concern about the methodological integrity of the recently published study by Kader et al. (7), “Joint effect of sleep duration and sleep quality on self-rated health among Canadian adults: estimating relative excess risk due to interaction from a nationwide survey.” It analyzed data from the Canadian Community Health Survey to assess joint effects of short sleep (< 7 h) and poor sleep quality on self-rated health, reporting an odds ratio (ORs) ~2.5 for poor health when both risk factors were present. The study has several methodological limitations that raise questions about the validity and interpretability of its conclusions.

In this commentary, we discuss three primary methodological concerns: limited generalizability, potential bias introduced by dichotomizing key variables, and interpretation challenges in using ORs and additive interaction measures in a cross-sectional study. Our aim is to clarify these concerns and provide constructive direction for strengthening future research in this important area of sleep and population health.

### 1.1 Representativeness and limited regional restriction

A major concern is the study's restriction to six provinces and territories, excluding Ontario and Manitoba, which together account for roughly 38% of Canada's population (8). Omitting these large and socio-demographically diverse provinces introduces substantial risk of selection bias and limits national generalizability, particularly if sleep patterns or health outcomes differ meaningfully across regions. The rationale for this exclusion was not provided, and although briefly noted in the discussion but no actions were taken, like sensitivity analyses or even discussing the justification for omitting these states. This is a major limitation of the study's generalizability.

### 1.2 Oversimplification of key variables of health and sleep measures

The study simplifies key variables in ways that obscure important nuances. The dichotomization of self-rated health into “Good or Better” vs. “Fair or Poor” reduces a five-point ordinal measure to a binary outcome, discarding meaningful variation in perceived health. The reason and methods of this dichotomization are not clear in the methodology section. Similarly, the categorization of sleep duration into “ < 7 h” and “≥7 h” fails to account for the non-linear relationship between sleep and health outcomes, particularly the adverse effects of long sleep duration. The binary coding of trouble sleeping further conflates transient and chronic sleep disturbances, potentially misclassifying exposure. Preserving the original scales or applying more refined categorizations would allow a more accurate characterization of the complex relationships between sleep and health outcomes. For sleep duration, we recommend treating it as a continuous variable with a non-linear term (such as a restricted cubic spline) (9), or at least distinguishing short, normal, and long sleep. This approach captures the known U-shaped association between sleep length and health outcomes (10), where both short and long sleep are linked to worse health.

### 1.3 Questionable use of RERI estimation and cross-sectional data

The authors estimated additive interaction using Relative Excess Risk due to Interaction (RERI) derived from odds ratios in a cross-sectional dataset. While RERI quantifies whether the joint effect of two exposures exceeds the sum of their individual effects (11), applying it to ORs is statistically and conceptually challenging. When outcomes are common in cross-sectional analyses, ORs overestimate associations (12) relative to risk ratios, diverging non-linearly from true probabilities and compromising interpretability (13). This limitation can be addressed using log-binomial or modified Poisson regression models with robust error variance, which provide valid estimates of risk ratios for additive interaction.

### 1.4 Ethical oversight in data governance considerations

Thus, the original study complied with established ethics norms for secondary data analysis (14). However, ethical responsibility extends beyond formal review requirements. Analyses involving sensitive health and behavioral variables should still acknowledge data governance principles, including privacy safeguards and the potential for re-identification. While anonymization substantially reduces risk, a brief statement on these considerations would strengthen transparency and reinforce research integrity.

### 1.5 Risk of misleading causal interpretations

The cross-sectional design of the CCHS inherently precludes causal inference; however, aspects of the paper's framing suggest directional or causal interpretations. Given that exposure and outcome were assessed concurrently, it is not possible to determine whether poor sleep precedes adverse health outcomes, whether the reverse is true (15), or whether both are influenced by unmeasured confounders. Presenting additive interaction effects without temporal or causal context risks misinforming policymakers and the public, potentially contributing to misdirected interventions, suboptimal resource allocation, and diminished confidence in sleep health research.

## 2 Conclusion

This study by Kader et al. draws attention to a significant public health issue but is undermined by methodological shortcomings. It demonstrates how methodological compromises and insufficiently justified analytic choices can produce imprecise or potentially misleading findings. Careful methodology using representative data, avoiding arbitrary dichotomization, and selecting appropriate risk measures is essential. Future research should employ designs that support causal inference and adopt modern analytical approaches, such as ordinal models or spline-based methods, to better capture complex dose–response relationships.
